# The views of people living with chronic stroke and aphasia on their potential involvement as research partners: a thematic analysis

**DOI:** 10.1186/s40900-022-00379-1

**Published:** 2022-09-05

**Authors:** Marina Charalambous, Alexia Kountouri, Phivos Phylactou, Ioanna Triantafyllidou, Jean-Marie Annoni, Maria Kambanaros

**Affiliations:** 1 Laboratory of Cognitive and Neurological Sciences, Faculty of Science and Medicine, University of Fribourg, Chemin du Musée 8, 1700 Fribourg, Switzerland; 2 Department of Rehabilitation Sciences, Cyprus University of Technology, 30 Arch. Kyprianos Str. 3036, Limassol, Cyprus; 3 Solidarity Network Nicosia In Action” (NicInAct), Multifunctional Foundation, Nicosia Municipality, Eptanisou 11, 1016 Nicosia, Cyprus; 4 Allied Health and Human Performance, University of South Australia, Adelaide, SA Australia

**Keywords:** Stroke, Patient and public involvement, People with aphasia, Thematic analysis, Impact, Co-produced research

## Abstract

**Background:**

Patient and Public Involvement (PPI) is the active partnership between researchers, patients and laypeople in the process of creating research. PPI in stroke aphasia research aims to ensure equal opportunities for informed decision-making and guarantee democratic representation of patient partners within the research team. Yet, little is known about the factors that hinder and/or promote the autonomous involvement of people with aphasia in stroke and aphasia PPI projects. This study aimed to explore the views and perspectives of people who live with chronic stroke, with and without aphasia, with experience in research prior to stroke, on their potential involvement as research partners.

**Methods:**

The research team included a PPI partner with chronic stroke-induced aphasia. Semi-structured interviews were conducted online with people with chronic stroke (n = 8), four with aphasia and four without. Interviews were subject to thematic analysis.

**Results:**

Inductive thematic analysis generated four themes: (1) the kinds of *Restrictions* that make involvement in research difficult, (2) the preferred levels and ways of *Involvement* during the research process, (3) the *Support* required for active and collaborative involvement*,* and (4) the *Impact* of their involvement and how it benefits the study’s outcomes.

**Conclusion:**

People experiencing chronic stroke and aphasia are willing to be involved as PPI partners if the research team provides the necessary support. Recommendations for researchers to consider before commencing co-produced research with people with stroke and aphasia are provided.

**Supplementary Information:**

The online version contains supplementary material available at 10.1186/s40900-022-00379-1.

## Plain English Summary

Patient and Public Involvement (PPI) is the active collaboration between researchers and patient groups when conducting research. PPI in aphasia research after stroke is growing in importance. Aphasia is the communication difficulty after stroke that negatively affects speaking and/or understanding, reading and/or writing. People with aphasia are usually involved in a tokenistic way in research teams. Tokenism is including people in the research process, who are usually at a disadvantage, by pretending that their involvement gives the appearance of equality. To avoid tokenism researchers must ensure equal opportunities for patient partners with communication challenges to contribute as PPI researchers. This requires a supportive environment, easy access to information and opportunities for making informed choices. This study aimed to explore the views of people who live with chronic stroke and aphasia on whether they would like to be involved in PPI projects and in what manner. All participants had experience of the research process prior to the stroke event. The research team included a person who lived with chronic aphasia after her stroke. She was named the PPI partner. Interviews were completed online with eight people with chronic stroke. Four with aphasia and four without. Interviews were analyzed and generated four themes (RISI): (1) the kinds of Restrictions patient participants face due to stroke, (2) their preferred levels of Involvement, (3) the Support they need for active and meaningful engagement and (4) their views on the Impact of PPI on research outcomes. The findings were mapped onto the International Classification of Functioning and Disability framework of the World Stroke Organization to depict how an individual’s health status, limitations in activity and environmental barriers for active participation influence their engagement within the research team. People living with chronic stroke, either with aphasia or without, are willing to be involved as patient partners. But researchers need to provide the necessary support suited to their communicative and other stroke-related needs. An aphasia inclusive PPI model will encourage meaningful research activities that make an impact, avoid research waste, rejuvenate the researcher-patient relationship dynamics, translate research outcomes into everyday life, and empower communities of people living with stroke and aphasia.

**Supplementary Information:**

The online version contains supplementary material available at 10.1186/s40900-022-00379-1.

## Introduction

Patient and Public Involvement (PPI) in stroke aphasia research is growing in importance and magnitude [1, 2]. PPI is the active collaboration between researchers, patients and laypeople in the process of creating research [3]. Recently, a moral line of reasoning was asserted highlighting the importance of democratic representation and the empowerment of people with aphasia (PWA) after stroke in research [2]. A strong argument for embracing the PPI model in stroke-induced aphasia research is that it will improve the quality, credibility and relevance of the study design, methods, results, and the impact on research or health outcomes [4]. The importance of measuring the impact of patient-orientated research is a crucial parameter of the patient partnership research model, and requires continuous monitoring and evaluation [5, 6].

The work of Staniszewska et al. on the development of the GRIPP (Guidance for Reporting Involvement of Patients and the Public) [5] and the revised GRIPP-2 [6], framed the significance of the impact of PPI on the research itself, the patient partners involved and society at large. Funding bodies have also embraced this expectation and request confirmation and transparency on how patients are engaged in the preparation of the application and their involvement and interaction in the research process [7]. Emphasis is also given to the value of experiential knowledge provided directly from the lived experiences of PWA after stroke [1]. This also has practical consequences concerning authorships, guided by journal publishing policies, that demonstrate authentic commitment to PPI, and robust research practice that is ethical [8, 9]. This study, is the first part of the PAOLI (People with Aphasia and Other Lay People Involvement) project that aims to develop guidelines for involving people with aphasia in qualitative participatory research.

Patient and Public Involvement projects encompass different participatory approaches [10]. An approach, that includes a range of different activities throughout the research cycle, can be defined in relation to ‘ways of doing’, ‘ways of knowing’’, and ‘ways of changing’. However, not all participatory approaches achieve the same level of participation [10]. In 1969, Sherry Arnstein published an influential paper entitled ‘A Ladder of Citizen Participation’. In this paper, Arnstein [11] described the steps involved in citizen participation metaphorically as a hierarchical step ladder beginning at the bottom rungs with non-participation, (i.e., ‘manipulation’) and moving up the ladder to ‘degrees of tokenism’, (i.e., ‘informing’ and ‘consultation’), and reaching the top rung of full citizen control (i.e., ‘partnership’ and ‘delegation’).

The general expectation is that PPI in stroke-induced aphasia research should underpin all stages of the research process, including how study topics are identified, prioritised, co-designed, conducted, interpreted, and disseminated [6]. McKevitt et al. [12] suggest that since stroke survivors do not perceive themselves to be an oppressed group, they have no strong desire to bring about social change, which deters them from being actively involved in citizen-controlled PPI research. This finding raises the question of what level or levels of participation are appropriate and feasible for PWA in stroke-induced aphasia PPI projects. In the study by Harrison and Palmer [13] a thematic analysis on the levels of PPI in stroke research, the authors recommend that prior to the involvement of people with stroke, researchers should be proactive and consider how representative of the specific projects the PPI members need to be and to reduce underrepresentation, whether experiences alone are sufficient for inclusion, and if PPI members need to have additional professional skills and training to promote their level of involvement.

Even though PPI in stroke-induced aphasia research aims at reducing tokenism by ensuring equal and ethical opportunities for involvement of PWA [14] the PPI concept remains problematic. It does not encompass the issues and complexities of involving patients with persistant communication impairment and/or other disabilities. In a recent scoping review, Charalambous and colleagues [15], explored whether PWA after stroke were involved as research partners in the development of Quality of Life (QOL) and aphasia-impact related questionnaires given the immediate relevance of both topics to their current condition. The findings revealed that PPI was mostly consultative in nature with insufficient or fragmented reporting of PPI contribution levels [16]. One main reason for placing PWA in a consultative role is because researchers do not have explicit resources, methodologies, and frameworks on how to train, work and support PWA throughout the research cycle [15]. Interpreting the findings in relation to Arnstein’s ladder of citizen participation suggests that researchers employ tokenistic engagement by predominantly *informing* PWA about the research topic and *consulting* with PWA on the relevance of the items on the pre-established measures and later deciding whether to act upon their suggestions or not (*placation*) [11].

For this study, the intention was to recruit people with stroke and aphasia, with prior experience in research, as we expected that their involvement with the research process and their living stroke experience would help them to anticipate the difficulties that PWA and stroke might face when involved in the PAOLI study. We also wanted to identify the kinds of support that would be needed by this group throughout the PPI process, their desired ways and level of involvement, their personal motivations, and their expectations of the impact of their engagement on the research itself, themselves as individuals, and the stroke community at large [6].

The aim was to analyse and compare the views and opinions of PWA and stroke survivors without aphasia (SSwoA) on PPI, to probe the factors that might hinder and/or promote their active involvement in future PPI projects and to present their perspectives on the impact of PPI on the research outcomes. The findings were mapped onto the domains of the International Classification of Functioning (ICF) [17], framework to determine the impact of the barriers and/or facilitators to PPI on the individual’s health status, activity, participation enablers and other personal and environment (contextual) factors that influence the patient’s level of engagement within the research team. This approach aims to shift the focus of PPI research attention to the interactive and evolving linkages connecting people with stroke and aphasia to their environment, the research findings, and the impact of the research on health outcomes and clinical practice.

## Methods

### Study design

This study followed a qualitative approach to investigate the perspectives of people with stroke and aphasia with regards to their potential involvement as research partners. Semi-structured interviews were carried out to prompt views and opinions from people with stroke and aphasia. For this study, the term ‘PPI partner’ will be used to reflect the constant commitment and engagement of a person with chronic stoke-induced aphasia thought the research process. The aim for collaborating with AK as the PPI partner, was to enhance the quality, transparency, and consistency of the evidence base for this study by involving her in all stages of the thematic analysis. For the monitoring of the PPI partner engagement, we followed the GRIPP-2 Short Form checklist [6] (see Table 8 in the “Appendix”).

For the preparation, design, implementation, and analysis of the interview data the consolidated criteria for reporting qualitative studies (COREQ) 32-item checklist were used [18]. Previous interview protocols used in aphasia and stroke research were taken into consideration for the methods employed at the interviews [19]. The topic guide was developed within the research team in collaboration with the PPI partner, AK, and was based on GRIPP-2 Guidelines [6] and the Critical Outcomes of Research Engagement (COREs) questionnaire [20].

### Research team

The research team involved a senior female speech and language therapist practising in stroke-induced aphasia rehabilitation and research (MC) who was the primary investigator and collected all the data, a certified speech and language therapist who was the second data analyst (IT), an experimental psychologist with experience in thematic analysis (PP) who was involved in research methodology, data management and analysis and the PPI partner (AK).

AK is a 32-year-old female, with mild-moderate anomic aphasia who experienced a stroke 5 years prior to the study. She holds a master’s degree in Research Methodologies and dropped out of her doctoral studies in Social Care (University of Sussex) after her stroke event. AK is the Stroke Ambassador of the Cyprus Stroke Association, and she currently works as a community social worker under the auspices of the European program “Solidary Network in Action” for the Municipality of Nicosia (Cyprus). Her involvement encapsulates the ‘citizen control engagement level’ [10], that is, from the conceptualization of the research idea (to ask other people with stroke and aphasia about their opinions), in the design (proposed one to one semi-structured interviews for information gathering she suggested communication strategies to be used by the interviewer (MC) during the interviews), for recruitment (she suggested three other participants with stroke), for the analysis (she was present during the creation of the thematic matrix and the finalization of the results (key themes) and the dissemination of the study results (she is a co-author on this paper as she contributed to the lay sections, the selection of the infographics and the editing of the research paper).

### Selection criteria

Although PPI does not require prior experience and knowledge of the research process, the purpose of the sampling was to recruit PWA and SSwoA, with prior experience in research, that could engage in the discussion on level of involvement and could anticipate the pragmatic issues around PPI while having a chronic condition. Having previous experience in PPI projects was not an inclusion criterion for this study and the interviewer was blind to such information.

The inclusion criteria for this study were as follows: (1) to have experienced a stroke, (2) to be in the chronic stage of stroke (> 6 months post-stroke) (3) to speak, understand, read, and write English post-stroke (4) to be socially active as confirmed from case history (5) to have at least one academic qualification, and (6) to have had previous research experience, either as a student or as a researcher. An additional inclusion criterion for PWA was to show evidence, from case history interview, of mild-moderate chronic aphasia. People with stroke and aphasia were excluded if they experienced comorbidity with additional conditions e.g., dementia, cancer, other degenerative diseases etc.

The Aphasia Severity Rating Scale (ASRS), of the Boston Diagnostic Aphasia Examination (BDAE) [21] was used to rate the severity of the observed language difficulties (cut-off score 4/5). Spontaneous speech samples were elicited during a 15-min semi-structured interview that comprised of four topics: the illness, previous/current occupation, family and housing, hobbies [22]. Aphasia severity was assessed by the interviewer using the ASRS to allow a classification based on fluency and intelligibility. Scores on the ASRS range from 0 to 5, with 5 indicating very mild aphasic symptoms (‘minimal discernible speech handicap’) and 0 revealing very severe non-fluent aphasia (‘no usable speech or auditory comprehension’).

### Participant recruitment

Since PPI is now used in studies worldwide, geographical diversity was achieved by recruiting participants from various organisations that engage in stroke and aphasia research across Europe. Specifically, participants were recruited from the Cyprus Stroke Association (n = 4), the French Association S’ Adapter- AVC et Aphasie (n = 1), the Portugal AVC Stroke Association (n = 1), the Norwegian Stroke Association (n = 1) and via a snowball effect as the PPI partner, AK, suggested two people with stroke from the Stroke Alliance for Europe consortium who proposed another one (n = 3). Participants were recruited over a 3-month period (October–December 2020).

The main researcher MC provided participants with written information prior to the interviews about the study and the participants involvement. According to the guidelines of Kagan and Kimelman [23] informed consent was received from people with aphasia after all information were provided in an accessible way to promote comprehension [24]. Consent forms (see Additional file 1 ) were sent electronically to all participants via email and the interview process began as soon as participants had signed and returned their informed consent forms. Interviews were audio and video recorded upon consent.

### Participants

Eight people with chronic stroke, four with concomitant aphasia and four without, met the inclusion criteria and provided written consent to participate. Participants were aged between 27 and 70 years old, with a range of education in years between 15 and 22 years. All participants had completed a research project during their studies or work commitments prior to the stroke event. Specifically, one participant with aphasia (J.J) was the primary investigator in several research projects throughout his academic career and two SswoA participants (M.M. & V.V.) are currently the primary investigators in research projects in their perspective fields. The remaining participants were familiar with research process because of prior experience for the completion of thesis work while studying at University. All participants had right hemispheric stroke, were discharged from rehabilitation and were actively involved in the community. Participants with aphasia had received speech and language therapy intervention in the past and were currently active in aphasia communication groups. The demographic characteristics of all participants are shown in Table 1.

**Table 1:** Demographic characteristics of the participants

Participant	Gender	Age	Country	Stroke type (hemiplegia)	ASRS (0–5)	Completed education	Research experience	Premorbid employment (return to work)	ADL-I	Marital status	Social circle and friends (hobby)	Stroke/aphasia group attendance
*People with aphasia (PWA)*												
A.A	Female	27	Cyprus	Hemorrhagic LH (Yes)	4	Doctoral	Thesis completion	Teacher (no)	No	Single	Yes (yoga)	Yes
L.L	Male	30	France	Ischemic LH (yes)	4	Masters	Thesis completion	Lawyer (no)	Yes	Rela/ship	Yes (running)	Yes
J.J	Male	63	Switzerland	Ischemic LH (yes)	5	Doctoral	Primary investigator	Academic (retired)	No	Rela/ship	Yes (music)	Yes
C.C	Female	44	Greece	Ischemic LH (no)	5	Masters	Thesis completion	Administrator (no)	Yes	Married	Yes (pilates)	Yes
*Stroke survivors without aphasia (SSwoA)*												
M.M	Male	58	Cyprus	Ischemic LH (yes)	N/A	Doctoral	Primary investigator	Academic (yes)	No	Rela/ship	Yes (poetry)	No
V.V	Female	55	Switzerland	Ischemic LH (no)	N/A	Doctoral	Primary investigator	Academic (yes)	Yes	Married	Yes (running)	No
G.G	Female	44	Denmark	Ischemic LH (no)	N/A	Bachelor	Thesis completion	Unemployed N/A	No	Married	Yes (knitting)	No
I.I	Female	40	Portugal	Hemorrhagic	N/A	Masters	Thesis completion	Nurse (yes)	Yes	Divorced	Yes (drawing)	Yes

LH, left hemisphere; ASRS, Aphasia Severity Rating Scale (0 = severe expressive aphasia, 5 = very mild aphasic symptoms); AADL-I, ctivities of daily living—independent

### Pilot interviews

The topic guide was pilot tested with the PPI partner AK, in practice interviews with the primary investigator MC. Due to the complexity of the subject, the PPI partner AK suggested to pilot again with a second person with aphasia who did not participate in this study. Two questions were excluded because of similarity in content and/or ambiguity.

### Data collection

The primary investigator MC conducted in-depth semi-structured interviews with all participants during a three-month period. From the start, an agreement between the interviewer and each interviewee was reached for the exact date and time at which the interviews would take place. The interviews were conducted via the online platform of Zoom. The first communication was via email. Specific instructions were provided to the interviewee to eliminate possible interruptions of the video call procedures (i.e., be in a familiar and comfortable environment, reduce environmental sound etc.). These were followed and maintained throughout the interview. MC monitored for signs of fatigue, emotional distress, and participants were encouraged to take breaks whenever required.

The main researcher MC employed a flexibility in the methods of the interviews to promote maximal inclusion for participants with communication and language challenges after stroke. Both the researcher and the participants were actively involved in composing meaning via dialogue. Following the constructivist paradigm as described by Guba and Lincoln [25], the personal views and opinions of the participants originated from co-construction via online interaction with the researcher MC [19].

The interviews were on average of 50 min duration and were conducted in English as both the interviewer and the interviewees were bilinguals. The interview began with a general discussion to explain the research procedure, to gather personal history data, and to build rapport. A smooth transition from pre-established open-ended questions to more specific ones was performed for easier categorisation of the qualitative data and reduction of bias. Since having previous experience in PPI was not compulsory for this study, broad questions assisted in initiating the discussion such as: “*Have you ever heard of studies with Patient Involvement as researchers?”, “Would you like to be involved in research as a partner? and in what way?”, “Why is important for you to be involved as a research partner?”, “In what stage of the research process would you like to be involved?”, “To what extent would you like to be involved?”, “How could the research team support you with written materials?”, “How could the research team support you with discussions?”, “How can you be involved in dissemination?”, “How do you think your involvement could influence the research outcome?”, “How will your involvement impact you as a person?”.*

Additional supplementary questions to allow scaffolding were: *“Do you prefer study materials to be simplified and written in a common/plain language?”, “How do you manage situations where you find it challenging to follow group discussions?”, “What kind of support would you need while reading complex paperwork?”, “Do you travel for meetings?”, “What is your main challenge after your stroke?*”, *“Do you think is important for other people with stroke and aphasia to know a study’s outcomes?”.* The topic guide facilitated semi-structured interviews without fixed sequencing or wording and used non-directed open questions where possible, allowing for scaffolding, including binary choice alternatives and yes/no questions where necessary. Scaffolding also included providing examples of what other people with stroke and aphasia had said in early interviews. This structure allowed MC to provide explanatory questions if needed. During the interviews, clarifications were made by the researcher when needed, so that participants could understand the content of the question and provide the necessary answers. The interviewer, MC, used strategies suggested by the PPI partner, AK, to ensure that significant others did not speak for participants with aphasia or interfere during the interview. Examples of her suggestions are as follows: “if the person is searching for a word, do not try to complete their sentence or suggest a word unless they specifically ask you”, “if the person starts to struggle in the conversation, do not interrupt but give them a chance to respond at their own pace”.

When the interviews were completed, the recorded videos were transferred to researcher IT for transcription. All data were transcribed verbatim by IT and were reviewed by MC. Before initiating the thematic analysis*,* an online meeting between the two experimental psychologists experienced in thematic analysis (author PP & colleague) and the two researchers (MC & IT), was performed to discuss the principles and methodology of the thematic analysis.

### Data analysis

**Fig. 1:**
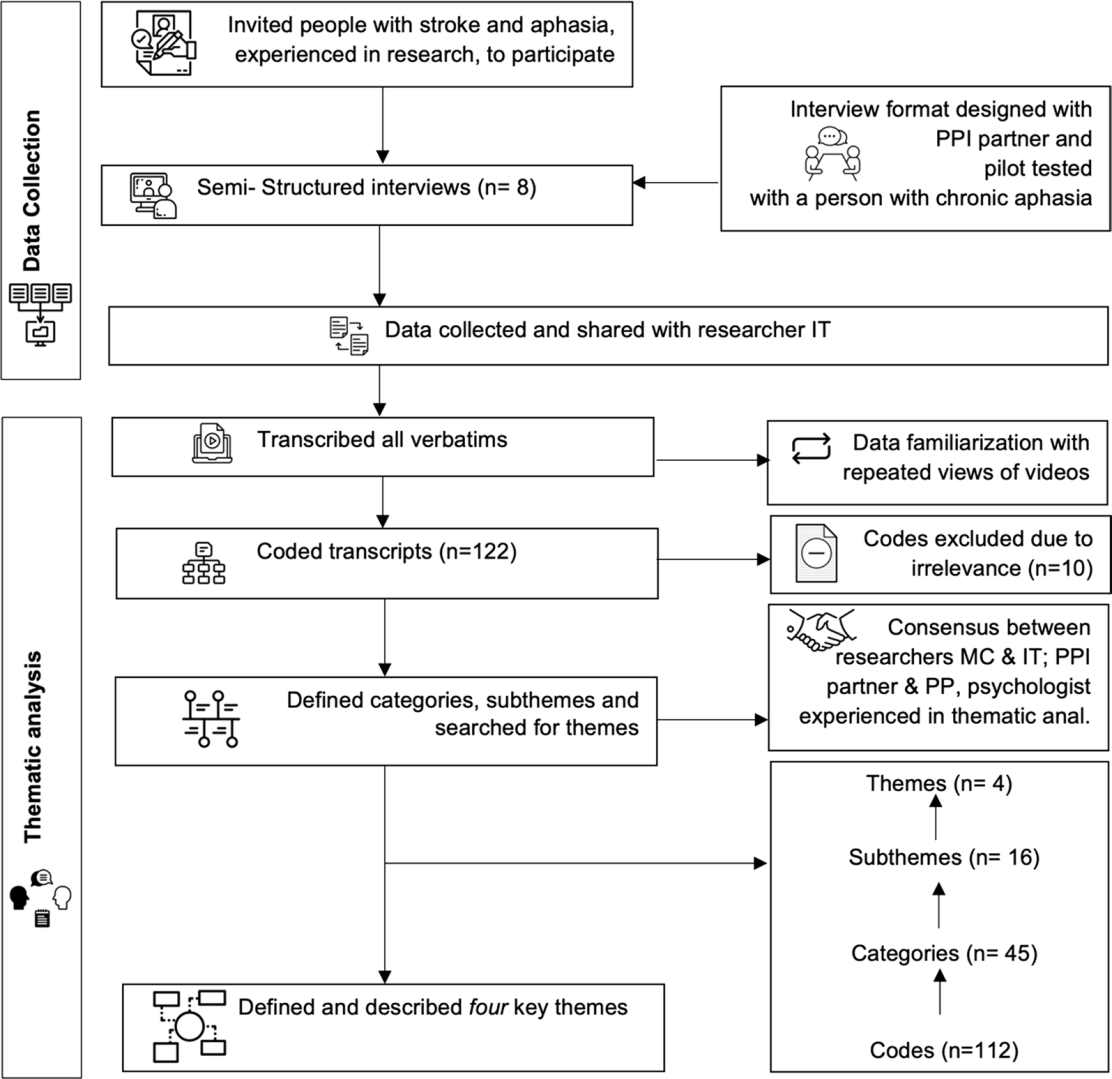
Flowchart of the thematic analysis processes

A thematic analysis was performed to gather and analyse a large quantity of information from participants into a meaningful account [26] following Braun and Clarke’s [27] 6-step framework:

- Step 1 *‘Familiarizing yourself with the data’:* The recorded data were viewed several times by both MC and IT (inter rater reliability). Over a period of three weeks all verbal data were transcribed into scripts and MC revised the transcripts again while watching each video separately (test retest). Later, all transcripts were again reviewed by MC and IT independently (intra evaluator coherence) and the final version of the transcripts was drafted. Non-verbal behaviours and cues (for example voice tone and volume, pauses, gestures, facial expression) pertinent to the research questions were documented in a separate file. All identifiable information was removed at transcription and participants were assigned random initials.
- Step 2 *'Generating initial codes’:* IT and MC proceeded with the initial generation of the raw codes. Codes are defined as a feature of the data that refer to “the most basic segment, or element, of the raw data or information that can be assessed in a meaningful way regarding the phenomenon” ([27], p. 63) e.g., ‘wheelchair’, ‘communication buddy’, ‘fatigue’. After initial code generation, the two researchers MC and IT had a face-to-face five-hour meeting with the experimental psychologist experienced in thematic analysis (author PP) and the PPI partner AK to initiate and monitor Steps 3, 4 and 5.
- Step 3 *‘Searching for themes’*: all codes with similar meanings were represented graphically in categories as discussed by each group. After codes were grouped into categories, sub-themes and finally themes emerged. A theme is defined as a word or a phrase that describes and summarizes the core point of a consistent and coherent idea patterned in the data [27] e.g., *Restrictions*. In the present study themes focused on the participants’ implicit ideas, perceptions, and views on their potential involvement in PPI research.
- Step 4 *'Reviewing themes’*: all initial themes were identified, examined, and differentiated to observe whether there was a connection between the data within the themes, while the differences between the topics were apparent to all four members of the research team. Themes were refined as part of an iterative process and consensus was reached between the four researchers (MC, IT, PP and AK). The final themes were then organized into a matrix, following a bottom–up approach, to generate a thematic map that represented the important information relayed in relation to the research questions.
- Step 5 *'Defining and naming themes’:* the selection of the names was dependent on the content and meaning of each theme. Again, all researchers concluded on names after consensus was reached. Finally, the thematic matrix with key themes, subthemes, and categories, was completed. When data analysis was completed, author MC proceeded with step (6) *‘Write-up’*. See Fig. 1 for the flowchart of the thematic analysis process.

### Rigor

Since qualitative analysis is becoming increasingly recognised and valued in research, it is vital that it is completed in a rigorous methodological manner to create meaningful results [28]. For this study the following strategies were employed to enhance the fidelity of the data: (1) pilot interviews were conducted which were evaluated within the research team, and ensured that the selected questions were accessible to PWA and lacked ambiguity [19, 29], (2) two online meetings took place to monitor the methods and procedures of the thematic analysis, (3) all transcripts were reviewed multiple times independently (test retest and for intra rater agreement) and (4) a face-to-face meeting took place to monitor the data analysis and interpretation.

## Results

**Fig. 2:**
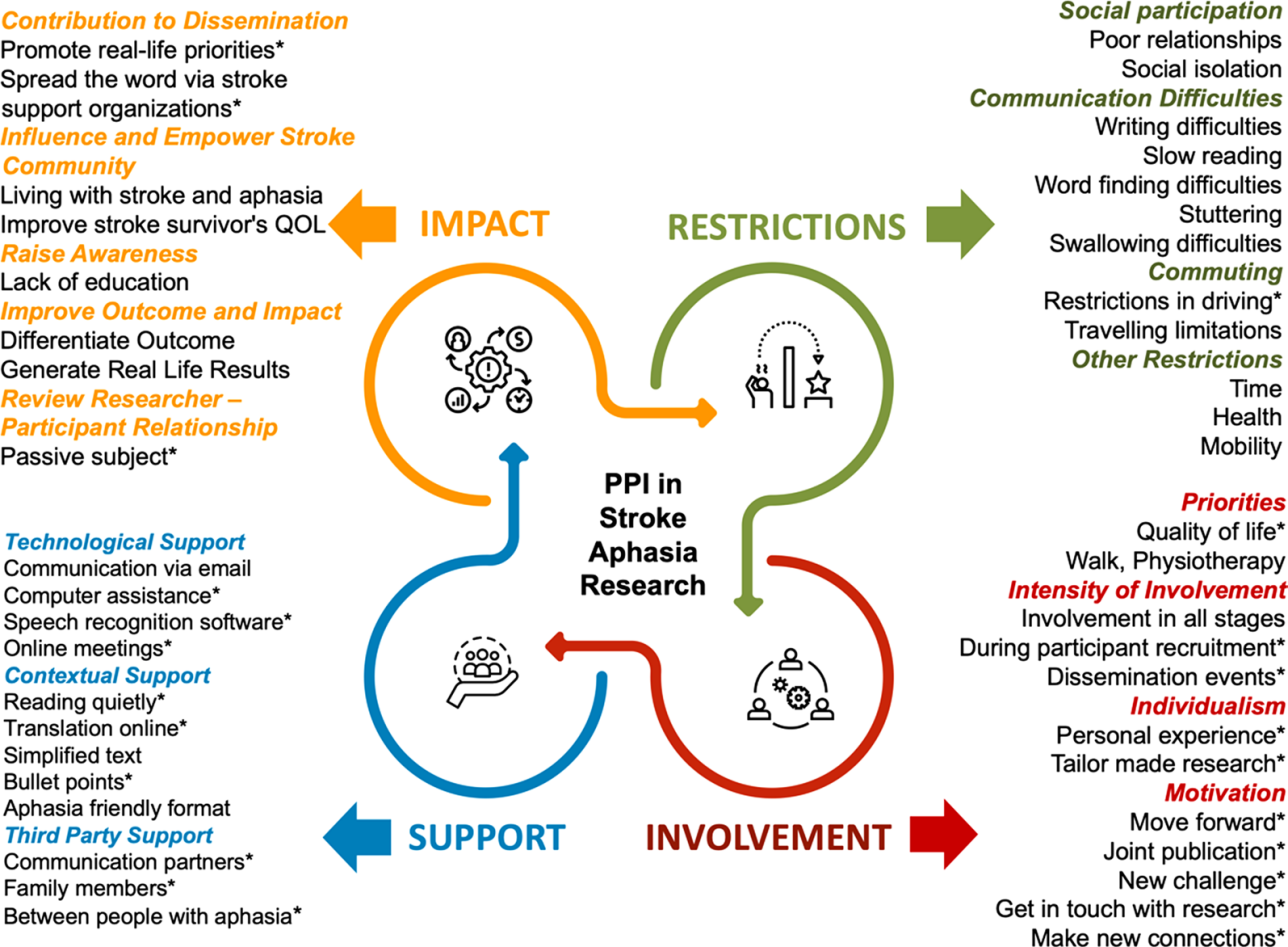
The thematic matrix with themes (n = 4), subthemes (n = 16) and categories (n = 45). *Discussed only by PWA

Initially, 122 codes were identified from the transcripts. Researchers MC and IT shortlisted the codes to 112. During the analysis, the bottom-up approach was used, starting from codes (n = 112) to categories (n = 45), from categories to subthemes (n = 16), and finally to key themes (n = 4) to result in a thematic matrix (see Fig. 2).

**Fig. 3:**
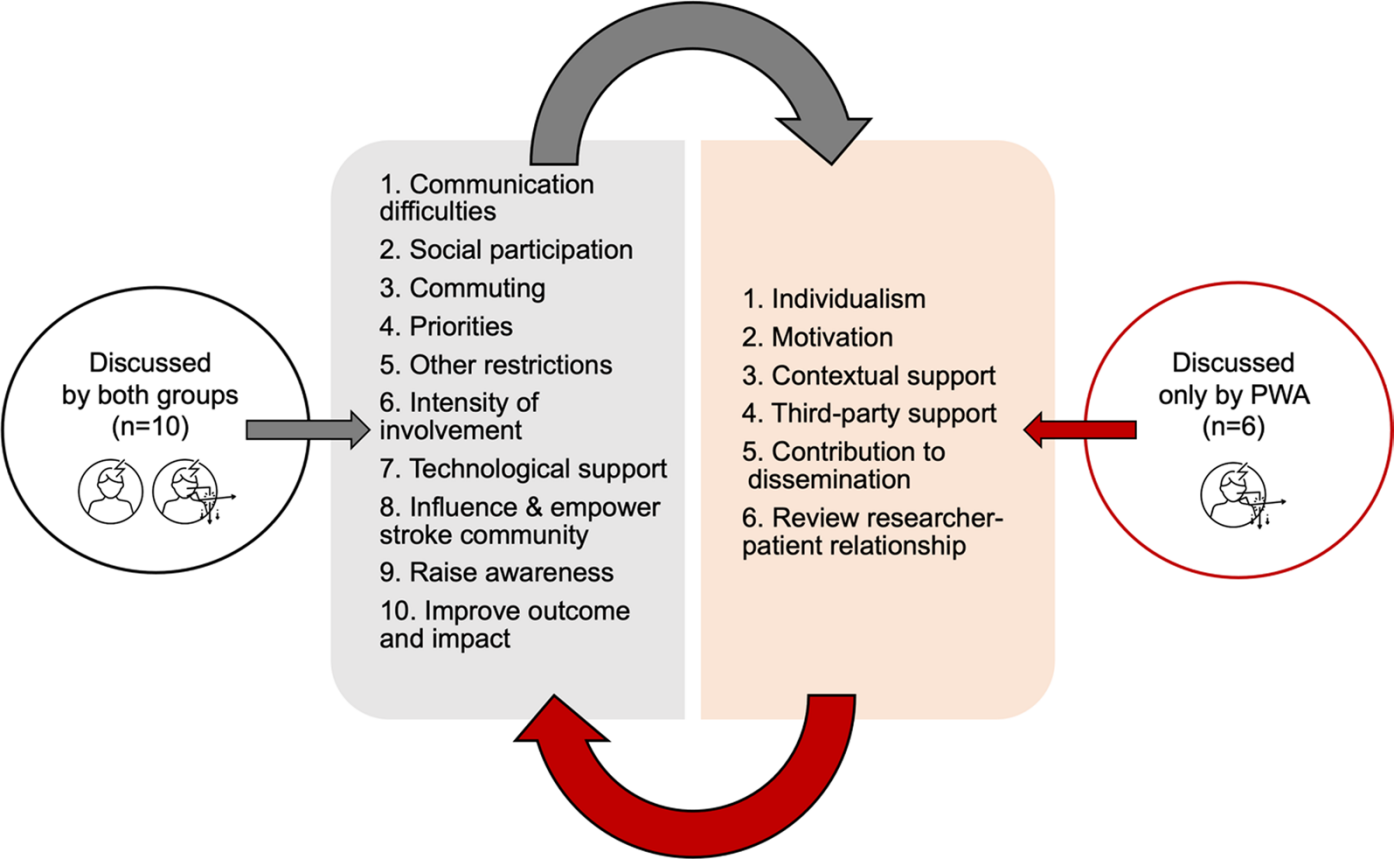
The subthemes (n = 16) as discussed by both groups and by the PWA group only

The four key themes were discussed by all participants. However, six subthemes (n = 6) were discussed only by PWA: (1) ‘Individualism’ and (2) ‘Motivation’ from the *Involvement theme*, (3) ‘Contextual Support’ and (4) ‘Third Party Support’ from *Support theme*, (5) ‘Contribution to Dissemination’ and (6) ‘Review Researcher—Participant Relationship’ from the *Impact theme*. Both groups mutually discussed 10 subthemes, with no subthemes discussed by the SSwoA group exclusively (see Fig. 3).

**Fig. 4:**
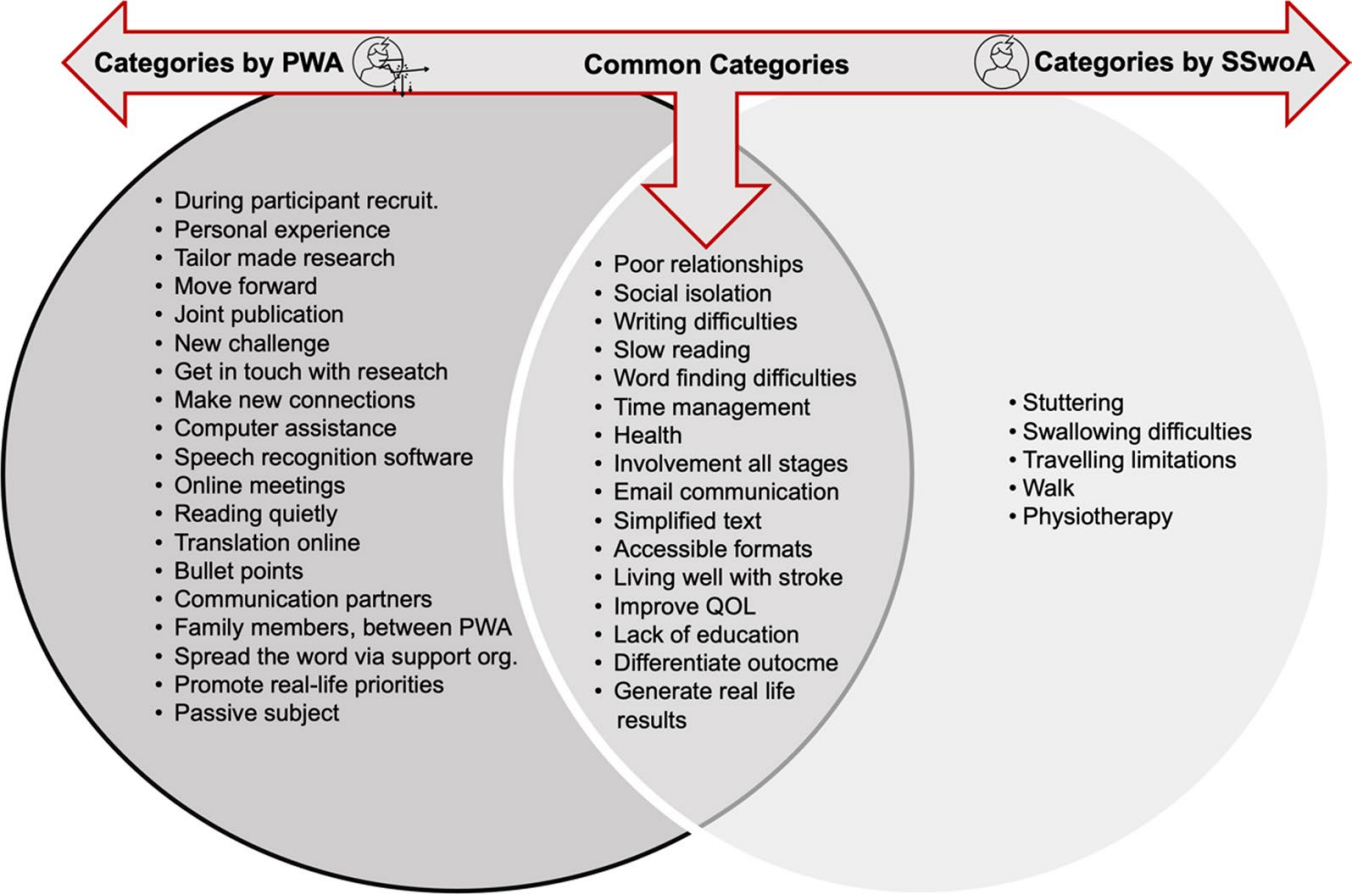
A Venn diagram reporting the categories discussed by PWA and SSwoA separately and together

For each group, individual categories have been represented in a section of the Venn Diagram. The categories discussed by each group individually and those discussed in common that resulted from the thematic analysis (see Fig. 4).

## The four themes

Four key themes emerged from the thematic analysis as follows: *Restrictions*, *Involvement, Support*, and *Impact*. *Restrictions* describes participants’ opinions about the difficulties they face after stroke that might prohibit their active engagement in the research process. *Involvement* portrays the ways in which participants would like to be included as members in the research team, whereas *Support* reports the approaches/methods that participants suggested can facilitate their active involvement and self-management throughout the research process. Finally, *Impact* reflects participants views on how they consider their involvement would benefit the research itself, but also the stroke and aphasia community.

It is important to mention that most participants (except I.I) responded negatively to the first question of the interview “Have you heard about studies with patient involvement as researchers?”. Most participants were surprised by the opportunity of such a methodological approach in health research. This observation is of high significance to researchers as it stresses the importance of raising awareness around PPI prior to patient partner recruitment. Even though most of the participants had some experience with research, either due to their professional occupations (researchers) and/or academic studies, they all requested a brief explanation from the primary investigator, MC, on the characterization of PPI and how it is reported in recent studies. Once MC provided examples of PPI in recent cancer and dementia studies [9, 30], participants began to be engaged with the interview topic. Both groups (PWA & SSwoA) discussed the importance of sharing personal ‘lived’ experiences post stroke with the rest of the research team to promote ‘realistic’ and ‘pragmatic’ research [31, 32].

### Theme 1: restrictions

The *Restrictions* theme includes participants’ opinions on the constrains and barriers they might face as PPI members of a research team, due to either their difficulties with communication and mobility, or other stroke-related health issues (see Table 2).

**Table 2:** Theme 1 results on ‘Restrictions’ with subthemes, categories, and examples

Theme	Description	
Restrictions	The kind of difficulties that make engagement in research challenging	
*Subthemes*	*Categories*	*Participant testimonies*
Social participation (3)	Poor relationships (3) Social isolation (2)	[I.I] “Like…And there were many people that tell me things like…. that I did not call you because I thought you could not speak…”
Difficulties (6)	Writing difficulties (5) Slow reading (3) World finding difficulties (3) Stuttering (1) Swallowing difficulties (1)	[L.L] “For example, when I write email, maybe it's a simple email 2 sentences or 5 sentences. And then I have to reread, reread, reread. And I have [short pause] write down the few words. The one sentence, if it's short sentences [short pause] long sentences […] I can write it but i i can i need to reread, reread, reread, reread, and I send my documents also my the mother of my girlfriend. And she checked the words the sentence. I guess I I have problem… I know, I know, don't have problems with sin… syntax. But when I write long sentence” [A.A] “ I having difficulty in participating in the group when writing something […] Yes yes.. eehh okay and if it’s a difficult, for example article, I can read them but I will need much more time” [L.L] “ [Discussing about group engagement] there is two problems. My aphasia and also my [short pause] I can say it in French [short pause] (Participant raises his hemiplegic hand to the screen)” [J.J] “[..] but you have just to know, that I'm not very good with typing. So, for me to return an email, maybe takes a day or two” [V.V] “When in a group you and you can’t remember the name of this also, or the name of this intellectual or precise things like this and its challenging…”
Commuting (2)	Restrictions in driving (1) Traveling limitations (2)	[M.M] “I haven’t travelled yet, and I don’t know if I would ever can do it [long pause] I don’t know, it’s difficult, I need support” [C.C] “I cannot take the car…nor the bus. It takes time [long pause] tired. Maybe do meetings online”’ [A.A] “I need someone to take me to places..with aphasia is difficult to drive [short pause] home visit is better for research”
Other restrictions (6)	Time management (6) Health (4)	[L.L] “And I don't think that I will be a partner [short pause] fully, fully, because my work and my work my, my [short pause] time” [M.M] “Time is always an issue. You see I have my lectures, research and family also. Maybe not full-time research partner” [V.V] “I think what is very difficult to me to be in group. I had the stroke when I was 40 and my kids were kids. And so, when I came back I was lucky that I had very light consequences, but I have to manage somehow my real state of fatigue. It’s difficult to attend long meetings… it makes me more tired”

In parenthesis is the number of participants that reported on each subtheme and subcategory

A significant topic under *Restrictions* were the *communication difficulties* because of stroke discussed equally by PWA and SSwoA. Specifically, SSwoA referred to acquired stuttering, difficulties with swallowing and writing challenges due to hemiplegia whereas PWA were focusing on the impact of aphasia. PWA explained their current communication difficulties across language modalities, and how their poor communication skills led to high risk of social isolation, and poorer social relationships and therefore low engagement with the PPI team. Also, PWA supported the view that their communication and language difficulties might pose a barrier to their contribution as research partners.

Moreover, participants discussed the issue of *commuting* as their reluctance to travel to other cities or countries for research purposes mainly because of low confidence in travelling alone or unattended. Likewise, *time* was an issue discussed extensively as stroke survivors felt unable to devote themselves entirely to research, due to personal and professional obligations. Additional limitations regarding their *health* were examined by both groups. Specifically, participants argued how hemiplegia, aphasia, depression, and fatigue, in combination or solely, could challenge their capacity to partake in the research team.

### Theme 2: involvement

The second theme *Involvement* emerged from the views of all PWA and two SSwoA on the reasons and the ways they would like to be included in the research team as partners (see Table 3).

**Table 3:** Theme 2 results on Involvement with subthemes, categories, and examples

Theme	Description	
Involvement	The preferred levels and ways of involvement during the research process	
*Subthemes*	*Categories*	*Participant testimonies*
Priorities (2)	Quality of life (1) Walk (2) Physiotherapy (1)	[A.A] “Life with a stroke is difficult, and with aphasia [long pause] I want better quality in my life [long pause] let people know”
Intensity of involvement (4)	Involvement in all stages (4) During participant recruitment (3) Dissemination events (4)	[A.A] “I want In the interviews, groups in research, do questionnaires, things like that..I can also invite C.. can also join the team” [G.G] “From beginning… until the end (Participant nods head for confirmation)” [I.I] “I can tell people from my group to join the team” [J.J] “I can send to my French aphasia group… your research the, the questionnaires also, the results”
Individualism (4)	Personal experience (3) Tailor made research (3)	[A.A] “ It will be helpful for the groups, aphasia groups, and to support the aphasia, aphasia group. For example, [short pause] let’s say you have a question, and they (the researchers) will tell their opinion and us our experience [short pause] they will improve the research question” […] Questions will be real, because people with the aphasia will be asking the question, and they know why they are asking” [J.J] Involved, that can bring something to the people with aphasia. It… ammm… making it easier for them to communicate? The… Yeah, sort of… I'd like to be involved…So that I can help them with whatever the problem is… a bit communicate, a bit the family, a bit at the workplace, whatever it maybe hopes to contribute a little bit to” [I.I] I think in some in some researchers, they do that (the PPI) and that's fantastic. Because it's completely different to understand the things or the person's point of view that knows exactly what they feel and also for the persons that are answering. If they feel understood, they open more. So some studies… some studies in health area do that, but not many. Not many that I know not that I'm aware” [C.C] “Questions will be real because people with the aphasia will be asking the question [short pause] and they know why they are asking [short pause] and they know that everybody else with aphasia will understand….”
Motivation (4)	Move forward (3) Joint publication (1) New challenge (1) Get in touch with research (3) Make new connections (4)	[A.A] I have one stroke in my life, and I would like to move forward. But myself…I will move forward…I would like to be more focused in stroke and aphasia research.”[…] “Experience. You will have more experiences. Compared with the ones who are not in these things” [C.C] “It is important …eem…yeah. I mean it’s… yeah I’m okay… I’m… I think so. It is actually challenging to be in research” [L.L] […] “And also, I would like to be more in research for stroke and aphasia. I don't know why, but [long pause]” (Participant raises both shoulders up)[…] “Because I was in Erasmus, I have friends all over Europe and I would, I would like to be more in touch with the research. I would like to be part of a European team” […] I would like to [short pause] go to see it and I would like to make connections for the aphasia groups”

In parenthesis is the number of participants that reported on each subtheme and subcategory

Under the *Involvement* theme participants argued that their intervention within the research team would be beneficial to guide the *research questions* towards the realistic needs of stroke and aphasia communities. Specifically, PWA discussed *individualism* and how through their *real-life experiences,* this will facilitate the formulation of specific questions and *tailor-made research*. With regards to the *intensity of their involvement,* participants stated that they wished to be involved in all stages of the research, from *recruitment* to *dissemination* since they have access to stroke and aphasia support groups. Finally, PWA stressed the importance of having a personal *motivation* to *move forward*, contribute to a research *publication,* experience a n*ew challenge, get back in touch with research,* and expand their social networks by m*aking new connections* with other people with stroke and aphasia.

### Theme 3: support

The third theme *Support* which involves opinions and views about the support mechanisms that will promote meaningful PPI within the research team was discussed by all PWA and one stroke survivor without aphasia (see Table 4).

**Table 4:** Theme 3 results on support with subthemes, categories, and examples

Theme	Description	
Support	The support required for active and collaborative involvement	
*Subthemes*	*Categories*	*Participant testimonies*
Technological support (3)	Online meetings (2) Communication via email (3) Computer assistance (3) Speech recognition software (1)	[G.G] “For example if we are deciding that we are going to do a questionnaire, do it online. Yes? Because it's different countries is difficult…. So we are not travel and we cannot meet. So we're doing online meetings” [C.C] “We can communicate with email. So I have time to respond, think and write” [L.L] “It's difficult to write…. I can write with my left hand. And, indeed. But it's more difficult. But I can write by computer” [J.J] “Because of the D.D program that that I have. With which, by the way, I used to do all the lectures and so on [short pause]. D.D is a speech recognition program. And it's a bilingual program. And so, it's always with English, English, German, English, French, English, Greek, and so on. And I dictate to the program […] the computer then types the text into the file”
Contextual support (3)	Reading quietly (1) Simplified text (2) Bullet points (1) Accessible format (2) Translate online (1)	[A.A] “I can read it slowly slowly, I mean quietly, and I use a highlighter and if I find difficulties, my mum or dad help me out to understand them or my siblings” [J.J] “Yes, short sentences would be easier for me” [L.L] “I can read. I can read without the images, the pictures. I can have a pattern that I would… that someone can help. I would like to…It's also difficult for me to understand in English. For me it's more or less bullet points” [C.C] […]“Pictures and simple words is help in the text” [A.A] “Emm when a find a word is unknown, I will search for it and I find its translation in Greek or English, online with a computer”
Third party support (4)	Communication partner (2) Family member (2) Between PWA (1)	[C.C] “I would like to be with someone with the English very good to help me with the team […] like the student in the aphasia group [short pause] to help with the group things to do in the team” [L.L] “I can write it but I I can I need to reread, reread, reread, reread, and I send my documents to my father, my girlfriend, my sister [short pause] to examine” [A.A] “People with aphasia can help other people with aphasia to understand and speak in the team”

In parenthesis is the number of participants that reported on each subtheme and subcategory

Participants discussed the need for constant *Support* to regularly attend face-to-face meetings due to their persisting difficulties with mobility, transportation, and communication. To reduce travel expectations, they suggested *online meetings* and *communication *via* email*. They also expressed that further *technological support* would be needed to promote functional communication such as the use of applications for direct messaging and communication related software. Moreover, PWA revealed the ways in which they can be facilitated to understand the research process with written material such as simplified text, bullet points, and the addition of pictures in line with the text. Additionally, PWA argued the need for constant third-party support from a communication partner, a family member or between PWA.

### Theme 4: impact

Finally, the fourth theme *Impact* was discussed by all participants. This theme included participants views regarding the impact they consider their involvement would have on the research outcome and for the stroke and aphasia community (see Table 5).

**Table 5:** Theme 4 results on Impact with subthemes, categories, and examples

Theme	Description	
Impact	The impact of their involvement and who it benefits research	
*Subthemes*	*Categories*	*Participant testimonies*
Contribution to dissemination (3)	Promote real life priorities (3) Spread the word via support organizations (3)	[M.M] “Involved, that can bring something to the people with aphasia. It [short pause] making it easier for them to communicate. Then yeah, sort of I'd like to be involved [short pause] so that I can help them with whatever the problem is a bit communicate, a bit the family, a bit at the workplace, whatever it maybe hopes to contribute a little bit to” [L.L] “Yeah yeah. Communications. But I would like to say it’s difficult for me and us in France. If you don’t speak English [short pause]” […] “I can send to my French aphasia group [long pause] to tell them” [J.J] if you invite me in conference or in workshop, I can either European one or French one, to explain the study”
Influence and empowering stroke community (4)	Improve QOL of people with stroke and aphasia (3) Living with stroke and aphasia (3)	[J.J] Improve the life of those living with the aphasia…that's the most important thing.. the outcome is so important for the people who have strokes and so on” [I.I] “I feel… I'm happy to do anything that can help people to understand the difficulties that persons with strokes or aphasia have, it's very important that other persons understand the difficulties and understand a little bit more about aphasia, so that people would noy want to be so isolated”
Raise awareness (2)	Lack of education (2)	[I.I] “Even health care professionals don't understand” […] “That [short pause] worries me very much is how much [short pause] lack of information professional health have [short pause] because I was I was Intensive Care Nurse. I was in the top I should know everything, and I didn’t [long pause]”
Improve outcome and impact (5)	Differentiate Outcome (4) Generate real life results (4)	[V.V] “I think aphasia is one of the hardest difficulties that people can have. And it's also one of the difficulties that isolate more the stroke survivors, especially the young ones. And, and I've been trying to call attention in the hospital about that, because…they are quiet, and they stay at home. And I think is one of the hardest sequels that persons can have. Because then they cannot express. So, it's really hard. So, it's… I'm very happy if we are doing something in that area” [J.J] “Real results from life… Yeah, that's, that's for sure… that's for sure. And I really hope that people are going to read it afterwards. Understand and change things…” [A.A] “Results will be different…people will ask the questions, so real outcomes. Will have more real outcomes” [C.C] “My part [short pause] it will have more experiences and more quality than…Yes research will have different and real result [short pause] better quality after”
Review researcher participant relationship (1)	Passive subject (1)	[C.C] “So I remember… because I was doing my myself for another research. I knew very little [short pause] and I have to give my consent that of course my data could be used for research material and just [long pause] I felt that I was [long pause] I was in an object in a study if you wish, so I didn’t feel that I was the agent in that [short pause]. I mean it was fine with me but, this is how I conceived the relationship between researcher and the patient. The patient is somehow by definition passive, I guess”

In parenthesis is the number of participants that reported on each subtheme and subcategory

Under *Impact* participants argued that their contribution to the research process will positively affect the *Dissemination* of the results to stroke communities due to their role as patient representatives. However, they stated that it might be challenging to disseminate research results to local stroke and aphasia societies since most research is written in English, a non-dominant language in their countries. Likewise, participants discussed the *Impact* of such an inclusive PPI partnership research model on the stroke and aphasia community in terms of positive *influence* and *empowerment.* They also debated the lack of education around stroke and aphasia, the wish to raise public *awareness* and for health professionals to promote *successful living with stroke and aphasia*. To *improve outcome and research impact* participants discussed that their involvement would generate *real life results* due to their original contribution. Lastly, a participant with aphasia emphasised the passive role of patients as subjects in research studies and discussed a must-needed change in the power dynamics of the *patient-researcher relationship* with the addition of patient partners in research teams.

## Discussion

This study, which to our knowledge is the first attempt to investigate the views and perspectives of people with chronic stroke, with and without aphasia, around potential involvement in PPI projects. All eight participants were experienced in research prior to their stroke, although this is not mandatory for participation in PPI studies. The anticipated challenges of active PPI in stroke and aphasia research and the support mechanisms that would promote the active, collaborative, and independent involvement of people with communication and/or physical and sensory impairments after stroke at the level of ‘citizen-control’ were reported. The results magnify the research impact of PPI for end users, the study itself as well as the stroke and aphasia community at large. Reflecting on the active engagement of AK as a PPI partner in this study had a positive influence in the following areas: AK’s involvement from the onset of the study helped the team formulate the research questions, she actively contributed to the study’s methodology, she reported a positive experience of her involvement in the study as researcher and felt well-supported, and she was a key player in the consensus meeting for the emergence of the four themes.

### Similarities between PWA and SSwoA

The findings of this study revealed that both groups contributed similar responses to the research topic guide, with little variation from PWA. Although none of the participants were familiar with the term PPI or had any previous experience, they all stated an interest in becoming involved in PPI studies under certain conditions. Both groups acknowledged the importance of the PPI partnership in stroke and aphasia studies as means of generating meaningful results. The most identified themes included information on how restrictions in mobility, barriers to transportation, communication difficulties and other health limitations are factors that hinder the frequency and intensity of their involvement. Participants from both groups stressed the importance of a robust support system within the research team governance, to encourage co-produced PPI research at the level of citizen-control.

The *key* theme *Support* incorporated additional strategies recommended by the participants on how they could be effectively supported by the researchers within the research team in managing reading, writing and group discussions. Both groups also discussed the issues related to the profound symptoms of stroke under *Restrictions* and how motor and communication difficulties pose barriers to their motivation for synergistic engagement within the research team [13, 33, 34]. *Restrictions* in commuting and time-management factors were also discussed. This was also true in the Harrison and Palmer [13] study where participants mentioned that distance and restrictions in travelling due to motor and access difficulties, restricted the frequency and intensity of their physical presence and hence overall involvement in the meetings of the research team. Time restrictions due to personal and professional engagements were similarly evident in the Harrison and Palmer [13] study where participants stated that during the active period of the study, they did not have enough spare time to get sufficiently involved. Thus, it is important that researchers explain to patient participants, before their involvement about how they will be safely transported to the research venue on every occasion and how they will be financially supported to use personal vehicles or transportation [35, 36]. Also, to eliminate the ‘burden’ of being involved in time consuming studies the research team should determine mutually agreeable timetables and research agenda timelines before the commencement of the research.

Similarly, the issues around *Involvement* discussed by both groups, highlighted how their acquired communication difficulties, beyond aphasia, impacted their social interaction skills which can impose on the collaboration process within the research team. Such difficulties can be monitored by encouraging PWA to attend weekly stroke and aphasia support groups that promote social connectedness and improvement of communication skills [37]. Different methodological approaches used in support groups such as learning events, personal stories, conversation strategies, and patient narratives can be used to foster communication skills practice and active engagement [1, 38]. Finally, prior to the recruitment of patient partners, researchers should proceed with a one-on-one meeting with each potential patient partner to build rapport, briefly explain their commitments and obtain a detailed medical history. Such an approach would enable researchers to be proactive and create an inclusive and positive research environment for PPI.

### Differences between PWA and SSwoA

Differences between the two groups emerged during a deeper analysis of the data. They highlighted individualism, the uniqueness of each person with stroke-induced aphasia, and the need to share personal perspectives and experiences about the impact of the loss of communication within the research team, as these are topics often ignored by researchers [39]. The results are in line with the evidence from other clinical populations that have communication impairment and difficulties with access to research involvement, for example, people with complex speech and motor disorders [40]. Discussion on motivation also emerged from the PWA group. PWA were able to identify several types of motivational factors to promote their involvement as follows:

- Establish new links to the stroke society by making new connections with other people with stroke and aphasia
- Extend on their previous research work
- Get in touch again with known past researchers
- Participate in a joint research publication
- Acquire a deeper understanding of their condition
- Move forward in terms of personal development by taking on the challenge of collaboration in PPI projects.

We also report participants’ testimonies that reveal socially motivated behaviour to engage in PPI studies, to raise stroke and aphasia awareness, not only in the stroke community but also in the public and the medical society.

Participants with aphasia also stressed the necessity for *constant third-party support*, i.e., communication partners and engagement from various facilitators to manage verbal and written content within research consortiums. Communication partners are assigned to each individual with aphasia, and their goal is to facilitate the understanding and communicative access (not necessarily verbal) for PWA within the research team [41]. Since all participants with aphasia were actively engaged in aphasia communication support groups, they could anticipate the value of communication partners as part of the research team. The recruitment of communication partners and facilitators such as healthcare students and trained volunteers [42–44] is warranted to avoid the risk of being tokenistic within the team. It is suggested that potential patient partners with aphasia be recruited from aphasia communication groups, as they are familiar with communication partner practices, are acquainted with group interactions and are more likely to express their individual needs and views during the research process.

People with aphasia also advocated for various communication facilitators under *technological support* to enhance the conventional methods of engagement such as speech recognition software, technological aids etc. They suggested information on *contextual support* such as use of simplified text, pictures, and infographics in written material which supports previous research on accessible material for people with aphasia [24]. Additional training sessions on accessible research vocabulary, design, and methodology before the initiation of the study [45] are recommended. Any such facilitators or compensation strategies should be defined, prepared, and monitored by researchers before patient groups become involved with the research process. Finally, PWA expressed the wish to be part of the dissemination process that could be realised via access to their stroke and aphasia support groups. They purported to be in a better position to communicate research findings and terminology in a less complicated manner that peers with aphasia and communication challenges would appreciate. The importance of contextual and third-party support was only stressed by PWA as a prerequisite for active engagement (citizen-control level of Arnstein’s ladder).

A critical key point discussed by the PWA group was the participant-researcher relationship. This emerged from a participant’s negative experience of being a passive subject in a study following her stroke. This area puts into question the traditional role boundaries, raises multiple ethical dilemmas and methodological challenges in qualitative and experimental research for people with communication impairments [2] and stresses the important topic of participation and power equalization in the patient-researcher relationship [31].

### The factors that hinder or promote the active involvement of PWA and SSwoA

The factors that hinder or promote the active involvement of PWA and SSwoA have been identified and are presented in Tables 6 and 7. Specifically, the factors that might hinder active engagement in potential PPI studies are mostly related to communication and commuting difficulties, poor social participation, and stroke related health issues (see Table 6).

**Table 6:**
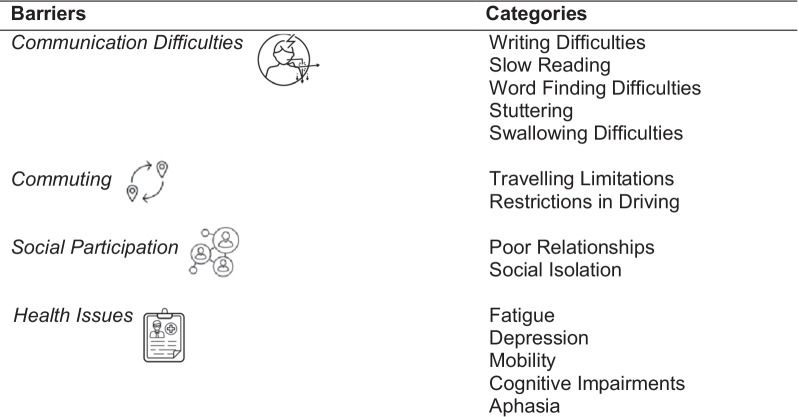
Barriers to active engagement

**Table 7:**
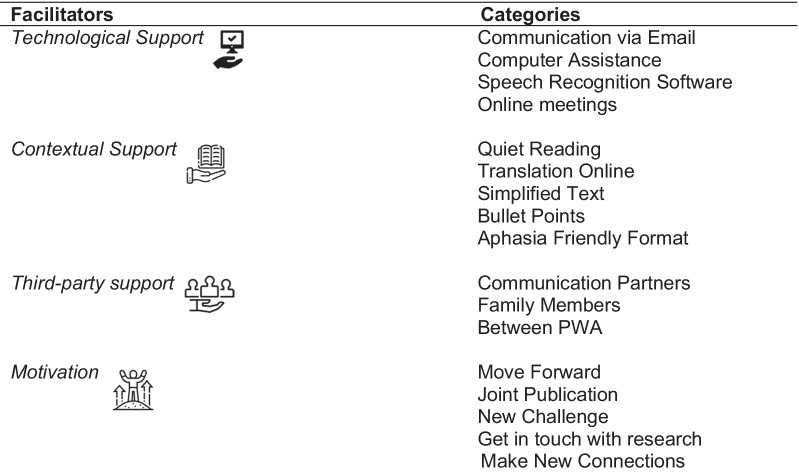
Facilitators that enable active engagement

Furthermore, the following facilitators were raised by all participants, such as technological, contextual and third-party support and motivation that might promote their engagement in citizen control PPI projects (see Table7).

### The impact of PPI

**Fig. 5:**
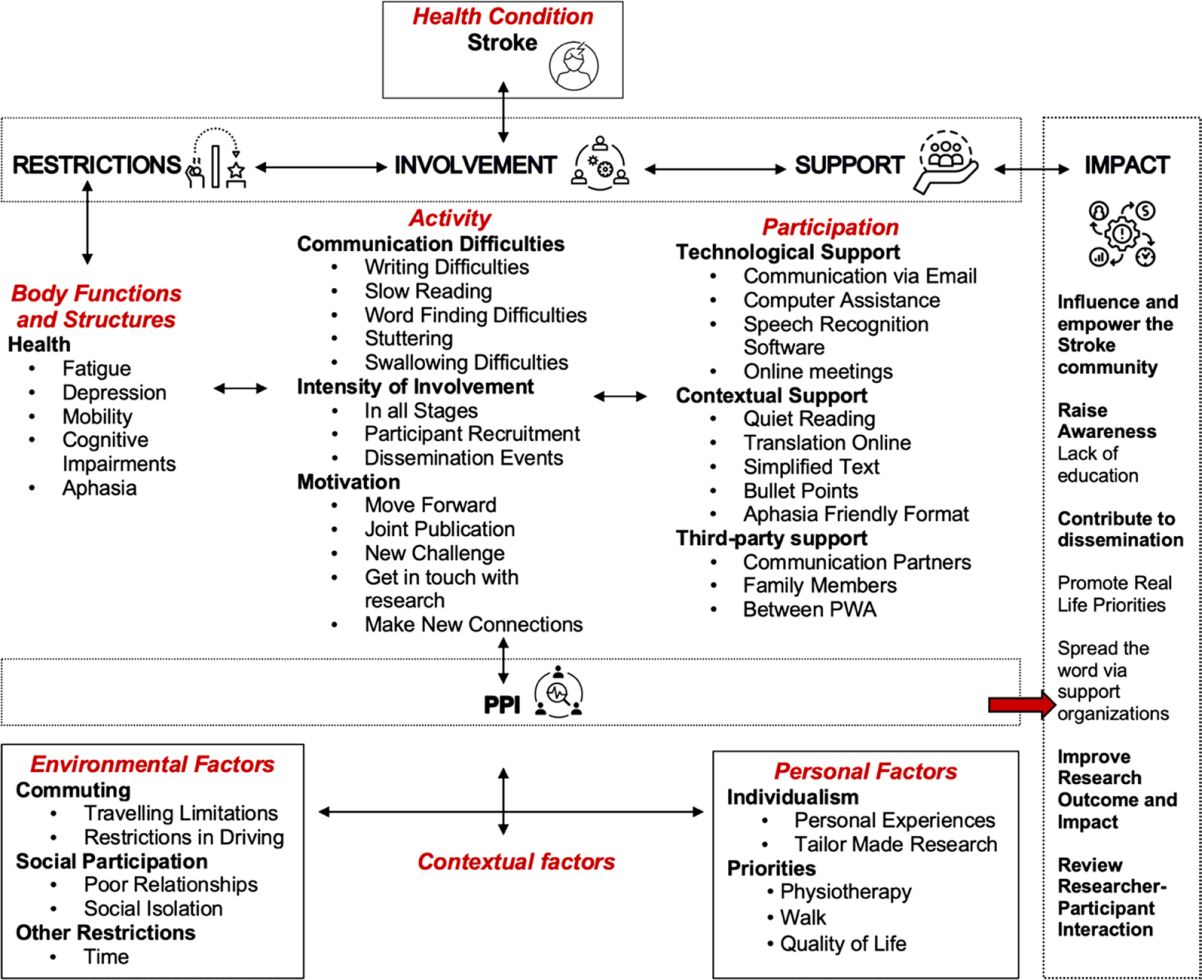
The themes (n = 4) and subthemes (n = 16) mapped onto the ICF

To conclude the discussion of the results, a second middle-ground approach was used based on the mapping of the results onto the ICF as a conceptual framework [17]. As predominantly a taxonomic scheme, the ICF provides a means of classifying variables associated with human functioning and disablement (body function and structure, activity, and participation) in context (environmental and personal factors). The mapping onto the ICF, which was completed by the research team after the analysis of the results, suggests focusing the PPI research attention, on the bi-directional and evolving linkages between people with stroke and aphasia to the environment, the research and the overall impact on the research process and outcomes [46]. This incorporates the four key themes, and all subthemes including the strategies that SSwoA and PWA suggest could implemented by the researchers for constant support during the PPI collaboration process (see Fig. 5).

Under the *Body functions and structure* domain of the ICF framework, the issues related to the profound symptoms of stroke, such as fatigue, spasticity, and aphasia are listed, and how these *Restrictions* are barriers and negatively influence the patient partners’ stimulation and collaborative engagement within the research team [34, 36]. Under the *Activity* domain the acquired communication difficulties are highlighted that restrict *Involvement* and personal motivation and scope to participate in such research will boost level of engagement*. Similarity,* under the *Participation* domain of the ICF framework, results were classified based on the *Support* needs proposed by participants. These include constant technological, contextual and third part support to boost communication effectiveness, paperwork management and meaningful engagements. Under the *Contextual factors,* which encompasses both *personal* and *environmental factors* affecting citizen-control PPI e.g., restrictions in commuting and time in contrast with personal motivation, priorities, and the need to express personal experiences for tailor-made collaboration processes. For example, the participants did not have to travel and were active participants so neither travel nor time reimbursement was organized for them.

The mapping of the PPI *Impact* as manifested by external variables and patient-centred factors onto the ICF framework, revealed the interface between stroke and aphasia with the overall impact of PPI on the research process. The impact of PPI, as discussed by participants, emphasises the immediate dissemination of the results to the public, aphasia and stroke support organizations, and the empowerment of stroke and aphasia communities. Through this practice, patients will be informed about stroke and aphasia and thus familiarize themselves with their condition, learn about living successfully with stroke and aphasia in the chronic stage and self-educate on self-management [47]. PWA also stressed that their involvement in PPI studies will promote stroke and aphasia awareness to the public, both for the acute stage but mostly for the ‘life after stroke’ phase where little or no evidence is available. Consequently, stroke and aphasia PPI endorses the demands of people living with stroke for better quality of treatment, rehabilitation, and community transition training that will strengthen the research impact. This should be considered as an important impact of PPI in stroke and aphasia research, albeit one that is in some ways distant from the pre-established tasks of reviewing patient information leaflets, commenting on pre-established questionnaires or assisting with recruitment processes.

This study underscores the value of the ICF as a conceptual tool in qualitative analysis of participatory research methods. Of potential merit is elaboration of the ICF in determining the personal factors and the provision of a context-driven, process view of person-environment interaction within PPI projects.

### Recommendations for PPI in stroke-aphasia studies

The findings of this study suggest that involving PWA in all stages of PPI research is challenging but possible. An inclusive PPI partnership model in stroke and aphasia research reaching the citizen control level of Arnstein’s ladder, will encourage important research endeavors to avoid research ‘waste’, invigorate the researcher-patient relationship dynamics, inform on the translation of the research into everyday life, and empower communities of people with stroke and aphasia. For these reasons, six recommendations for researchers to consider before commencing the research process with PWA were identified. The BEFORE recommendations are as follows:

1. Build rapport, offer information on PPI, and briefly explain research project commitments prior to recruitment including timetabling and the issue of transport
2. Establish communication needs and barriers to participation based on detailed information from the participant’s case history and interview, to prepare for research support accordingly
3. Foster a robust support system with communication partners, communication facilitators or compensatory strategies
4. Offer training courses on research vocabulary, design, and methodology using accessible aphasia-friendly formats
5. Reinforce the use of tailored technological and contextual resources
6. Encourage potential PPI partners to participate in stroke and aphasia support groups to practice communication skills and social connectedness. This will later put them in a better position to disseminate the research findings.

### Limitations

A limitation of this study is that no participant had previous experience with PPI projects and therefore the results cannot be generalized to all PWA and SSwoA to serve as research partners. Additionally, participants were active academics, former researchers, individuals who had experience in research methodology and could understand research practices and terminology. In this study we aimed to document the different opinions and views according to the diverse levels of exposure in research (participants were recruited if they had a university degree and research or therapeutic experience). Nevertheless, this can also be reported as a limitation as participants had dissimilar experiences in research projects. Some had minor experience in research, i.e., the completion of a bachelor’s thesis, compared to those who had completed doctoral studies, and had a vast experience in the research processes, which might not fully address the aim for this inclusion criterion.

For this study participants presented with mild to moderate chronic aphasia and were competent to independently participate using verbal communication. Since the interviews were exclusively carried out using an online platform, opportunities to use a variety of conversation support techniques to promote communication access were limited. Instead, the interviewer often repeated words or phrases to verify the interviewee’s understanding, incorporated closed questions, used simplified language, and clarified the subtle meanings of participant gestures or facial expressions. Also, because the interviews were semi-structured, participants often strayed from the topic of discussion, and directed the discussion to other topics, more personal and not related directly to the proposed subject. Additionally, the number of participants might be considered as small. However, with reference to saturation and the pragmatic considerations of this study, the number of participants is considered sufficient for the purpose of this research [48].

Finally, although the overall experience of AK as a PPI partner was positive, she reported a main concern at the end of the study. AK reflected that during the consensus meeting, because of the large volume of spoken and written material, she often experienced fatigue, and frequent breaks were necessary. A key challenge for the main researcher MC in respect to the involvement of a person with aphasia as a research partner was to prepare all written materials in accessible formats and allow time for AK to identify ideas and respond at her own pace. These factors made the procedure timely and very laborious for all researchers.

## Future recommendations

PPI research on stroke and stroke-induced aphasia is limited and has not been discussed at length in the literature. Our results revealed that the PPI partner role is complex and difficult to define, especially when people with stroke present with persisting communication difficulties. This might explain why representatives of people with stroke and especially those with aphasia, tend to be involved primarily in a consultative role rather than co-researchers in related studies. Future research needs to focus on the ways PWA can be supported throughout the research process. It is vital to generate guidelines and frameworks as guiding principles for the PPI partnership model, which will describe step by step, the methods and ways in which PWA can be facilitated to connect with the research process and prevent drop out. The BEFORE recommendations are a first step in this direction. Based on the findings of this study, the creation of such a conceptual framework will direct researchers on how to actively engage stroke survivors with aphasia in all stages of the PPI research process by monitoring its multidimensional nature and providing the necessary infrastructure to support it. In future research, the participants of this study will be re-interviewed after being involved as PPI partners in the PAOLI study, to explore change in their perspectives.

## Conclusion

In this study we have highlighted the invaluable role of PPI in stroke aphasia research and provided researchers practical recommendations to guide the involvement of people with chronic stroke, and especially those with aphasia, before the commencement of the research. All participants confirmed that they are willing to be involved in stroke and aphasia PPI studies but under specific conditions. This study generated novel findings about how PPI could be made accessible to PWA, presented the unique challenges, discussed the requirement for the development of robust support systems for a successful and sustainable PPI partnership model in stroke induced aphasia research and reflected the impact of meaningful contributions to research practices, end users and stroke and aphasia communities. It is hoped that this article will stimulate further discussion of the factors influencing the collaboration of people with stroke and aphasia in PPI projects.

### Supplementary Information

**Additional file 1. **Sample consent forms.

## Data Availability

Representative sample of transcript verbatims are presented in the article. The data generated during or analysed in the current study are not publicly available due to ethical restrictions. All data queries and requests should be submitted to the corresponding author, Marina Charalambous PhD Researcher, for consideration.

## Appendix

See Table 8.

**Table 8:** The GRIPP-2 short form

Section and topic	Item	Reported on page number
1. Aim	Report the aim of PPI in the study	8
2. Methods	Provide a clear description of the methods used for PPI in the study	9–10, 17–18
3. Study results outcomes	Report the results of PPI in the study	16–18
4. Discussion and conclusions on outcomes	Outcomes—comment on the extent to which PPI influenced the study overall	32, 38–39
5. Reflections/critical perspective	Comment critically on the study, reflecting on the things that went well and those that did not	45

PPI information reported on the GRIPP-2 SF

## Abbreviations

PPI: Patient and public involvement
PWA: People with Aphasia
GRIPP: Guidance for Reporting Involvement of Patients and the Public
QOL: Quality of life
PAOLI: People with aphasia and other lay people involvement
ICF: International Classification of Functioning and Disability Framework
COREs: Critical outcomes of research engagement
MC: Marina Charalambous
IT: Ioanna Triantafyllidou
PP: Phivos Phylactou
AK: Alexia Kountouris
ASRS: Aphasia Severity Rating Scale
BDAE: Boston Diagnostic Aphasia Examination
SSwoA: Stroke survivors without aphasia

## References

[1] Cruice M, Aujla S, Bannister S, Botting N, Boyle M, Charles N, Dhaliwal V, Grobler S, Hersh D, Marshall J, Morris S, Pritchard M, Scarth L, Talbot R, Dipper L (2021). Creating a novel approach to discourse treatment through coproduction with people with aphasia and speech and language therapists. Aphasiology.

[2] Hersh D, Israel M, Shiggins C (2021). The ethics of patient and public involvement across the research process: towards partnership with people with aphasia. Aphasiology.

[3] National Institute of Health Research (NIHR). Patient and public involvement in health and social care research: a handbook for researchers by research design service London; 2014. London: NIHR.

[4] Thompson J, Bissell P, Cooper C, Armitage C, Barber R (2013). Exploring the impact of patient and public involvement in a cancer research setting. Qual Health Res.

[5] Staniszewska S, Brett J, Mockford C, Barber R (2011). The GRIPP checklist: strengthening the quality of patient and public involvement reporting in research. Int J Technol Assess Health Care.

[6] Staniszewska S, Brett J, Simera I, Seers K, Mockford C, Goodlad S, Altman DG, Moher D, Barber R, Denegri S, Entwistle A, Littlejohns P, Morris C, Suleman R, Thomas V, Tysall C (2017). GRIPP2 reporting checklists: tools to improve reporting of patient and public involvement in research. BMJ.

[7] O’Donnell M, Entwistle V (2004). Consumer involvement in research projects: the activities of research funders. Health Policy.

[8] Mader LB, Harris T, Kläger S, Wilkinson IB, Hiemstra TF (2018). Inverting the patient involvement paradigm: defining patient led research. Res Involv Engagem.

[9] Swarbrick CM, Doors O, Scottish Dementia Working Group, Educate, Davis K, Keady J. Visioning change: co-producing a model of involvement and engagement in research (innovative practice). Dementia (London). 2019;18(7–8):3165–72. 10.1177/1471301216674559 10.1177/1471301216674559 27753612

[10] Tembo D, Morrow E, Worswick L, Lennard D (2019). Is co-production just a pipe dream for applied health research commissioning? An exploratory literature review. Front Sociol..

[11] Arnstein SR (1969). A ladder of citizen participation. J Am Inst Plann.

[12] McKevitt C, Fudge N, Wolfe CDA (2010). What is involvement in research and what does it achieve? Reflections on a pilot study of the personal costs of stroke. Health Expect.

[13] Harrison M, Palmer R (2015). Exploring patient and public involvement in stroke research: a qualitative study. Disabil Rehabil.

[14] Broomfield K, Craig C, Smith S, Jones G, Judge S, Sage K (2021). Creativity in public involvement: supporting authentic collaboration and inclusive research with seldom heard voices. Res Involv Engagem.

[15] Charalambous M, Kambanaros M, Annoni JM (2020). Are people with aphasia (PWA) involved in the creation of quality of life and aphasia impact-related questionnaires? A scoping review. Brain Sci.

[16] Pisano F, Marangolo P (2021). Editorial: new perspectives and methodologies in the diagnosis and rehabilitation of aphasia. Brain Sci..

[17] World Health Organization. The international classification of functioning, disability and health. 2001. Geneva: Author.

[18] Tong A, Sainsbury P, Craig J (2007). Consolidated criteria for reporting qualitative research (COREQ): a 32-item checklist for interviews and focus groups. Int J Qual Health Care.

[19] Lawton M, Haddock G, Conroy P, Serrant L, Sage K (2018). People with aphasia’s perception of the therapeutic alliance in aphasia rehabilitation post stroke: a thematic analysis. Aphasiology.

[20] Dillon EC, Tuzzio L, Madrid S, Olden H, Greenlee RT (2017). Measuring the impact of patient-engaged research: how a methods workshop identified critical outcomes of research engagement. J Patient Cent Res Rev.

[21] Goodglass H, Kaplan E, Barresi B. Boston diagnostic aphasia examination, 3rd edn. Boston: Pearson; 2000. p. BDAE-3.

[22] El Hachioui H, Lingsma HF, Van de Sandt-Koenderman MW, Dippel DW, Koudstaal PJ, Visch-Brink EG (2013). Long-term prognosis of aphasia after stroke. J Neurol Neurosurg Psychiatry.

[23] Kagan A, Kimelman M (1995). Informed consent in aphasia research: myth or reality. Clin Aphasiol.

[24] Rose TA, Worrall LE, Hickson LM, Hoffmann TC (2011). Aphasia friendly written health information: content and design characteristics. Int J Speech Lang Pathol.

[25] Guba EG, Lincoln YS, Denzin NK, Lincoln YS (1994). Competing paradigms in qualitative research. Handbook of qualitative research.

[26] Boyatzis RE (1998). Transforming qualitative information: thematic analysis and code development.

[27] Braun V, Clarke V (2006). Using thematic analysis in psychology. Qual Res Psychol.

[28] Nowell LS, Norris JM, White DE, Moules NJ (2017). Thematic analysis: striving to meet the trustworthiness criteria. Int J Qual Methods..

[29] Lloyd V, Gatherer A, Kalsy S (2006). Conducting qualitative interview research with people with expressive language difficulties. Qual Health Res.

[30] Miqueu P, Williams A, Kairenius A, de Valeriola D (2019). Patient involvement strategies to improve the quality of cancer care and research. Int J Integr Care.

[31] Isaksen J (2018). Well, you are the one who decides”: attempting shared decision making at the end of aphasia therapy. Top Lang Disord.

[32] Mann C, Chilcott S, Plumb K, Brooks E, Man MS (2018). Reporting and appraising the context, process and impact of PPI on contributors, researchers and the trial during a randomised controlled trial - the 3-d study. Res Involv Engagem.

[33] Towfighi A, Ovbiagele B, El Husseini N, Hackett ML, Jorge RE, Kissela BM, Mitchell PH, Skolarus LE, Whooley MA, Williams LS, American Heart Association Stroke Council; Council on Cardiovascular and Stroke Nursing; and Council on Quality of Care and Outcomes Research. Poststroke depression: a scientific statement for healthcare professionals from the American Heart Association/American Stroke Association. Stroke. 2017;48(2):e30–43. 10.1161/STR.0000000000000113 10.1161/STR.0000000000000113 27932603

[34] Schöttke H, Gerke L, Düsing R, Möllmann A (2020). Post-stroke depression and functional impairments—a 3-year prospective study. Compr Psychiatry.

[35] Maddox L, Doran DR, Hubbart G. Guide for researchers working with patient and public involvement (PPI) contributors. University of Oxford, University of Oxford’s Nuffield Department of Primary Care Health Sciences. 2017. London: National Institute of Health Research (National Institute on Handicapped Research).

[36] Stocks SJ, Giles SJ, Cheraghi-Sohi S, Campbell SM (2015). Application of a tool for the evaluation of public and patient involvement in research. BMJ..

[37] Berg K, Isaksen J, Wallace SJ, Cruice M, Simmons-Mackie N, Worrall L (2020). Establishing consensus on a definition of aphasia: an e-Delphi study of international aphasia researchers. Aphasiology..

[38] Kambanaros M (2019). Evaluating personal stroke narratives from bilingual Greek-English immigrants with aphasia. Folia Phoniatr Logop.

[39] Taubner H, Hallén M, Wengelin Å (2020). Still the same? Self-identity dilemmas when living with post-stroke aphasia in a digitalised society. Aphasiology.

[40] Jayes M, Moulam L, Meredith S, Whittle H, Lynch Y, Goldbart J, Judge S, Webb E, Meads D, Hemsley B, Murray J (2021). Making public involvement in research more inclusive of people with complex speech and motor disorders: The I-ASC project. Qual Health Res.

[41] Charalambous M, Kambanaros M. The importance of Aphasia Communication Groups. In: Jianu DC, Mureșanu DF, editors. Aphasia compendium [Internet]. London: IntechOpen; 2021 [cited 2022 Jul 24]. Available from: https://www.intechopen.com/chapters/79482. 10.5772/intechopen.101059

[42] Menger F, Morris J, Salis C (2017). Internet use in aphasia: a case study viewed through the international classification of functioning, disability, and health. Top Lang Disord.

[43] Simmons-Mackie N, Raymer A, Cherney LR (2016). Communication partner training in aphasia: an updated systematic review. Arch Phys Med Rehabil.

[44] Souchon NM, Krüger E, Eccles R, Pillay BS (2020). Perspectives of working-age adults with aphasia regarding social participation. Afr J Disabil.

[45] Caute A, Woolf C (2016). Using voice recognition software to improve communicative writing and social participation in an individual with severe acquired dysgraphia: an experimental single-case therapy study. Aphasiology.

[46] Duggan C, Albright K, Lequerica A (2008). Using the ICF to code and analyse women's disability narratives. Disabil Rehabil.

[47] . Rhoda A, Groenewald R, Altigani R, Jones F. Self-management and stroke. 2021. 10.1007/978-3-030-69736-5_5.

[48] Vasileiou K, Barnett J, Thorpe S, Young T (2018). Characterising and justifying sample size sufficiency in interview-based studies: systematic analysis of qualitative health research over a 15-year period. BMC Med Res Methodol.

