# Supplementary figures and images for: The views of people living with chronic stroke and aphasia on their potential involvement as research partners: a thematic analysis
Source: Res Involv Engagem. 2022 Sep 5;8:48. doi: 10.1186/s40900-022-00379-1 (PMC9446531; doi:10.1186/s40900-022-00379-1)

**Supplementary Material**

1. Typical Consent form

**
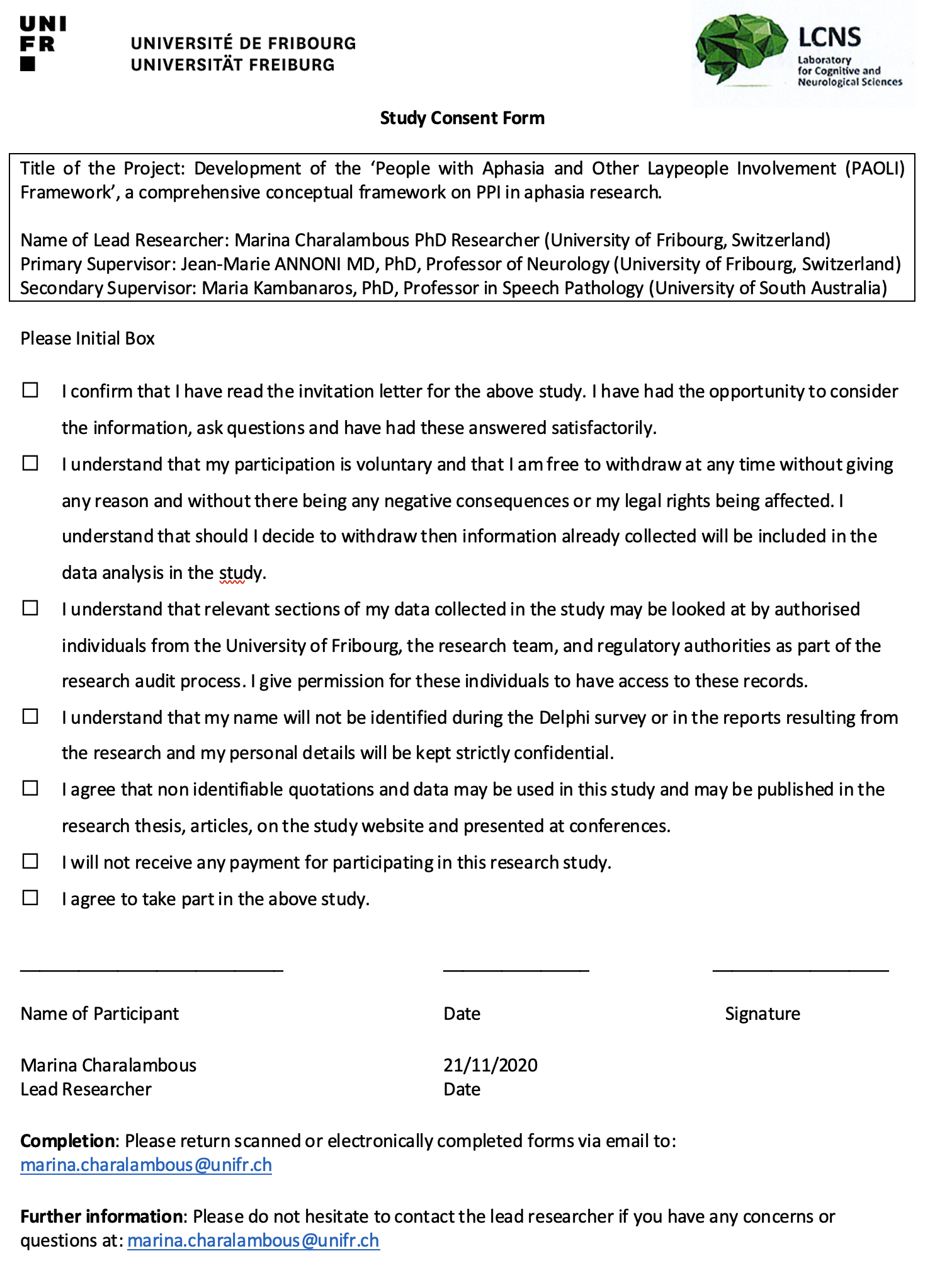
**

1. Aphasia Friendly Consent Form

**
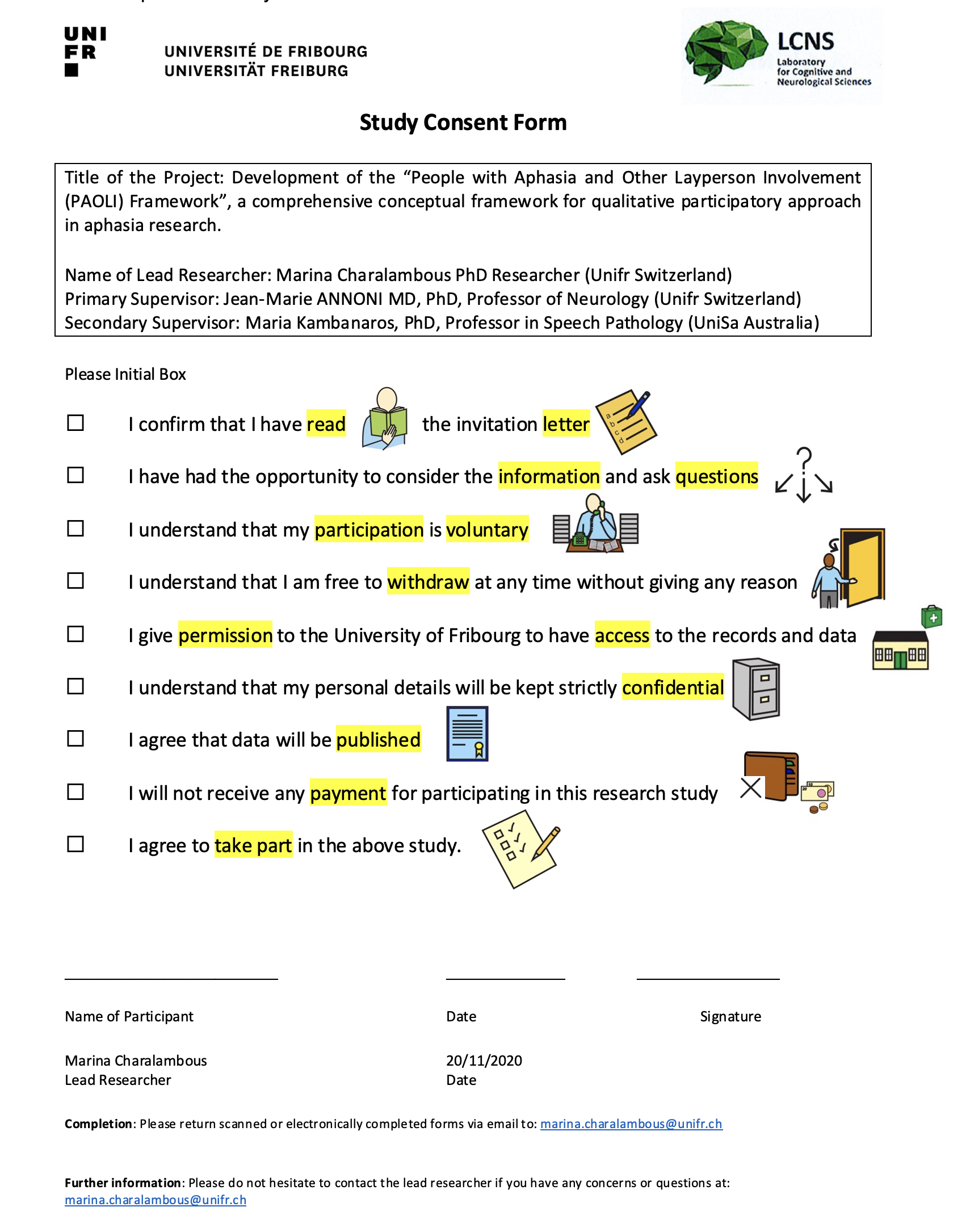
**

Supplement: Supplementary file 1 — Additional file 1. Sample consent forms. [file 40900_2022_379_MOESM1_ESM.docx]
